# Ruxolitinib plus standard of care in severe hospitalized adults with severe fever with thrombocytopenia syndrome (SFTS): an exploratory, single-arm trial

**DOI:** 10.1186/s12916-024-03421-z

**Published:** 2024-05-20

**Authors:** Sai Wen, Nannan Xu, Lianhui Zhao, Lulu Yang, Hui Yang, Caiyun Chang, Shanshan Wang, Chunmei Qu, Li Song, Wenlu Zou, Yishan He, Gang Wang

**Affiliations:** 1 Department of Infectious Disease, Qilu Hospital, Cheeloo College of Medicine, Shandong University, No. 107 Wenhuaxi Road, Jinan, 250012 Shandong Province P. R. China; 2 Jinan Center for Disease Control and Prevention, Jinan, 250021 Shandong China

**Keywords:** Ruxolitinib, Severe fever with thrombocytopenia syndrome, Cytokine storm

## Abstract

**Background:**

Severe fever with thrombocytopenia syndrome (SFTS) is an emerging tick-borne infectious disease, and its morbidity and mortality are increasing. At present, there is no specific therapy available. An exacerbated IFN-I response and cytokine storm are related to the mortality of patients with SFTS. Ruxolitinib is a Janus kinase (JAK) 1/2 inhibitor that can block proinflammatory cytokines and inhibit the type I IFN pathway. We aimed to explore the use of ruxolitinib plus standard of care for severe SFTS.

**Methods:**

We conducted a prospective, single-arm study of severe SFTS. We recruited participants aged 18 years or older who were admitted to the hospital with laboratory-confirmed severe SFTS and whose clinical score exceeded 8 points within 6 days of symptom onset. Participants received oral ruxolitinib (10 mg twice a day) for up to 10 days. The primary endpoint was 28-day overall survival. The secondary endpoints included the proportion of participants who needed intensive care unit (ICU) admission, total cost, changes in neurologic symptoms and clinical laboratory parameters, and adverse events (AEs) within 28 days. A historical control group (HC group, *n* = 26) who met the upper criteria for inclusion and hospitalized from April 1, 2021, to September 16, 2022, was selected and 1:1 matched for baseline characteristics by propensity score matching.

**Results:**

Between Sep 16, 2022, and Sep 16, 2023, 26 participants were recruited into the ruxolitinib treatment group (RUX group). The 28-day overall mortality was 7.7% in the RUX group and 46.2% in the HC group (*P* = 0.0017). There was a significantly lower proportion of ICU admissions (15.4% vs 65.4%, *p* < 0.001) and total hospitalization cost in the RUX group. Substantial improvements in neurologic symptoms, platelet counts, hyperferritinemia, and an absolute decrease in the serum SFTS viral load were observed in all surviving participants. Treatment-related adverse events were developed in 6 patients (23.2%) and worsened in 8 patients (30.8%), and no treatment-related serious adverse events were reported.

**Conclusions:**

Our findings indicate that ruxolitinib has the potential to increase the likelihood of survival as well as reduce the proportion of ICU hospitalization and being tolerated in severe SFTS. Further trials are needed.

**Trail registration:**

ChiCTR2200063759, September 16, 2022.

**Supplementary Information:**

The online version contains supplementary material available at 10.1186/s12916-024-03421-z.

## Background

Severe fever with thrombocytopenia syndrome (SFTS) is an emerging tick-borne infectious disease that was first reported in the Hubei and Henan Provinces of China in 2009 [1]. Subsequently, SFTS were reported in many other regions, such as Japan, South Korea, Vietnam, Thailand, and other provinces in China [2–4]. The pathogen of SFTS is SFTS virus (SFTSV), newly renamed Dabei Ribavirus, which is an RNA virus classified into the genus *Bandavirus* in the family Phenuiviridae [5]. The clinical manifestations of SFTS include acute fever with thrombocytopenia, leukopenia, gastrointestinal symptoms, and multiple organ failure, for which the mortality rate is as high as 32.6% [6]. Previous studies have reported that patients older than 60 years suffer hemorrhagic manifestations and multiple organ dysfunction accompanied by invasive pulmonary aspergillosis (IPA) and cytokine storms, which are associated with a greater risk of death [7, 8]. Li H et al. reported a clinical scoring model that showed good predictive power for a fatal outcome [9]. According to this model, a score greater than 8 points had 78% sensitivity and 82% specificity in predicting a fatal outcome. At present, there are no licensed vaccines or specific therapeutics available for preventing or treating SFTS [10]. Symptomatic treatment and supportive therapy are the most essential parts of management; although their efficacy remains limited [11, 12], new therapies are needed.

To date, the pathogenesis of the rapid progression of SFTS symptoms and the fatal outcomes of severe patients have not been well described. In several studies, a dysregulated host response and cytokine storm occurring in the acute phase of SFTS were associated with the development of multiple organ failure and poor outcomes, as in other viral infections [7, 13, 14]. It has been reported that there is a strong type I interferons (IFN) response in the peripheral blood mononuclear cells of patients with SFTS and COVID-19, which plays a pivotal role in exacerbating hyperinflammation in fatal SFTS as well as severe COVID-19 [14, 15]. In this respect, antagonists of proinflammatory cytokines, including those involved in type I IFN pathways, such as Janus kinase (JAK), might be potential treatments for blocking cytokine storms and improving outcomes in fatal SFTS patients.

Ruxolitinib is a JAK 1/2 inhibitor that can reduce the production and activity of proinflammatory cytokines by blocking JAK activity. Ruxolitinib has the potential to cause drug-induced liver damage, impair hematopoiesis, and create uncertainty regarding infection control in patients with myelofibrosis [16]. Several studies have reported promising results and safety for the use of ruxolitinib for the treatment of cytokine storms in patients with viral infections, especially COVID-19 [17, 18]. However, the clinical outcome of ruxolitinib in SFTS is still unclear and further investigation is needed. Therefore, we performed a prospective, open-label, single-arm investigation to explore the use of ruxolitinib plus standard of care for the treatment of severe hospitalized SFTS.

## Methods

### Study design and participants

Between September 16, 2022, and September 16, 2023, we conducted a prospective, open-label, single-arm study to assess the use of ruxolitinib combined with standard of care for the treatment of severe hospitalized SFTS at the Qilu Hospital of Shandong University (Additional file 1: Fig. S1). This single-arm study was designed according to CONSORT guidance [19].

Eligible participants were those aged 18 years or older who had been hospitalized with laboratory-confirmed SFTS and whose clinical score (Additional file 1: Table S1) was more than 8 points (for a total of 18 points) within 6 days of symptom onset with a high risk of death [9]. The age and neurological symptoms together with laboratory variables that were associated with death including abnormal concentrations of lactate dehydrogenase, aspartate aminotransferase, blood urea nitrogen, and abnormal neutrophil percentage were combined in this clinical scoring system. The laboratory-confirmed SFTS criteria used the definition released by the National Health Commission of China [10]: suspected diagnosed SFTS (an exposure history of previous field activities in SFTS-endemic areas or tick bites within 2 weeks before febrile symptom onset, acute fever with thrombocytopenia and/or leukopenia) with detection of SFTSV RNA by reverse-transcriptase PCR (RT-PCR). Participants (< 18 years) were ineligible, as were pregnant or lactating females. Other exclusion criteria included a history of ruxolitinib allergy, or unlikely survival longer than 24 h from enrollment in the investigator’s judgment. The detailed eligibility criteria are shown in Additional file 1 (page 2). Treatment suspension criteria included (a) the voluntary decision of the patient and (b) the treating physician’s decision to discontinue the treatment. All participants signed consent forms.

A historical control (HC) group of hospitalized SFTS patients who met the upper criteria for inclusion in our department was selected from April 1, 2021, to September 16, 2022. The HC group was matched 1:1 with the RUX group for baseline characteristics by propensity score matching (PSM).

The protocol was approved by the institutional review board of Qilu Hospital of Shandong University (KYLL-202206–011-1) and was registered at chictr.org.cn (number ChiCTR2200063759, September 16, 2022). All subjects provided informed consent before the procedure. This study was conducted in accordance with the principles of the Declaration of Helsinki.

### Procedures

After enrollment, participants received 10 mg (if the estimated creatinine clearance < 30 ml/min, 5 mg) of ruxolitinib (JAKAFI™) twice per day orally or through a nasogastric tube. The course of ruxolitinib treatment should be up to 10 days or until discharge from the hospital, whichever occurs first. All participants were allowed to receive the standard of care for SFTS treatment [10], which can include corticosteroids, plasma exchange therapy, intravenous immunoglobulin, and supportive treatment, such as antimicrobial agents, nutritional support, treatment of damaged organs, and mechanical ventilation support, according to the National Health Commission of China. Combinations of standard treatment protocols are determined by physicians based on the patient's condition.

The baseline measurements were recorded before the first dose of ruxolitinib was administered. Efficacy was evaluated for all participants by overall survival up to day 28, symptoms, physical examination, and laboratory monitoring up to day 14. Participants were scheduled for a follow-up visit 28 days after taking their first dose of ruxolitinib.

### Safety

According to the drug inserts and literature reports [16], the commonly observed adverse effects (AEs) caused by ruxolitinib administration were recorded up to day 28 and mainly included symptoms and abnormal measurements of laboratory parameters. The severity of the adverse events was evaluated by the investigators according to the National Cancer Institute Common Terminology Criteria for Adverse Events, v5.0 (CTCAE 5.0) [20]. Treatment-related AEs (TRAEs) were determined by three experienced physicians, referring to the drug inserts and previous literature reports.

### Outcome

The primary endpoint was overall survival up to day 28. The secondary endpoints included the proportion of participants who needed admission to the ICU, duration of hospitalization, total cost during hospitalization, dynamic changes in viral loads, and changes in laboratory abnormalities between baseline and posttreatment at days 3, 7, and 14. The adverse effects caused by ruxolitinib administration were recorded during the whole hospitalization and follow-up to day 28 and included symptoms and abnormal measurements of laboratory parameters.

Exploratory laboratory analyses of serum cytokines (interleukin-6 (IL-6), interleukin-8 (IL-8), interleukin-10 (IL-10), interferon-α (IFN-α), interferon-γ (IFN-γ)) and transcripts of the interferon-induced protein 44-like (IFI44L) gene (Additional file 1: P3) were performed before ruxolitinib treatment.

### Statistical analysis

We kept this exploratory study open to ensure that it was complete. The sample in this exploratory study included 26 participants up to September 16, 2023. We did not calculate the sample size due to the nature of single-arm studies.

To balance significant differences in baseline characteristics between the RUX group and the HC group, PSM was used to mitigate potential confounding factors (Additional file1: Table S2). Propensity scores were estimated for all patients through a multivariable logistic regression model, with groups as the dependent variable. Covariates included gender, time from initial symptoms to hospitalization, clinical score within 6 days of onset, symptoms, pre-existing comorbidity, and part of laboratory examinations including absolute platelet count, ferritin concentration, c-reactive protein, alanine aminotransferase, and creatinine. A 0.02 caliper was used for 1:1 matching.

Continuous data are presented as the means ± standard deviations (SD) (if normally distributed) or medians and interquartile ranges (IQR) (if not normally distributed). Comparisons of categorical variables were performed using the *χ*^2^ test or Fisher’s exact test. The independent *t* test and Wilcoxon-Mann‒Whitney test were used for normally and nonnormally distributed continuous variables, respectively. The Kaplan‒Meier curve was used to describe the overall survival of patients in each group, and the log-rank test and hazard ratio (HR) from the Cox proportional hazard model were used for time-to-event analyses. Statistical analyses were conducted using SPSS version 26 (Inc. Chicago, IL, USA) and GraphPad Prism (version 8.3.1 for Windows; GraphPad Software, www.graphpad.com). Statistical tests of treatment effects were performed at a two-sided significance level of 0.05, unless otherwise stated. A *P* value < 0.05 was considered to indicate statistical significance.

## Results

Between September 16, 2022, and September 16, 2023, we recruited 26 participants at Qilu Hospital of Shandong University. No participant withdrew their informed consent. The HC group was composed of 26 participants who met all the selection criteria and were hospitalized in our hospital before the initiation of this single-arm study. A total of 52 participants were evaluable for the major endpoint and parts of the second endpoint.

### Baseline characteristics

The baseline characteristics of these 52 participants are summarized in Table 1 (baseline characteristics before propensity score matching methods are summarized in Additional file1: Table S3). In the RUX group, the mean (± SD) age was 65.8 ± 8.7 years, and 57.7% of the patients were male. The mean time from initial symptoms to hospitalization was 7.2 ± 1.5 days. Seventeen (65.4%) of them had underlying conditions: 11 (42.3%) had a history of diabetes, and 6 (23.1%) had a history of hypertension. At the initiation of treatment, all participants had fever and at least one neurological symptom. Digestive symptoms, such as nausea and vomiting, were present in 8 (30.7%) individuals, while respiratory symptoms, including coughing and expectoration, were observed in 11 (42.3%) individuals. Six (23.1%) participants experienced bleeding symptoms that manifested as skin ecchymosis. The median platelet count was 45.0 (IQR 40.3–65.3) × 109/L. The mean (± SD) viral load was 6.2 ± 1.5 log10 copies/ml. At baseline, 24 (92.3%) participants had combined pneumonia according to computed tomography (CT) examination, and 19 (73.1%) participants had concomitant proven or probable IPA; probable IPA must include at least one host criterion, one clinical criterion, and one mycological criterion. Proven IPA was defined independently of the presence or absence of host, clinical, or mycological criteria. For further details, please see reference [21].

**Table 1:** Baseline characteristics in two groups

	**Total (*N* = 52)**	**RUX (*n* = 26)**	**HC (*n* = 26)**	***P*-value**
**General**				
Gender, male (*n*, %)	29 (55.8%)	15 (57.7%)	14 (53.8%)	0.78
Age (years)	64.6 ± 7.7	65.8 ± 8.7	63.4 ± 6.4	0.25
Time from initial symptoms to hospitalization (days)	7.4 ± 1.6	7.2 ± 1.5	7.7 ± 1.6	0.34
**Clinical parameters**				
Body temperature (℃)	38.8 ± 0.5	38.8 ± 0.5	38.8 ± 0.5	0.73
Gastrointestinal symptoms	18 (25.0%)	8 (30.8%)	10(38.5%)	0.56
Respiratory tract symptoms	20 (38.5%)	11 (42.3%)	9 (34.6%)	0.57
Neurologic symptoms	52 (100%)	26 (100%)	26 (100%)	1.0
Hemorrhage symptoms	13 (25.0%)	6 (23.1%)	7 (26.9%)	0.75
Clinical score within 6 days of onset	12.0 ± 1.7	12.2 ± 1.7	11.8 ± 1.8	0.78
**Pre-existing comorbidity**	34 (65.4%)	17 (65.4%)	17 (65.4%)	1.0
Hypertension	14 (26.9%)	6 (23.1%)	8 (30.8%)	0.53
Diabetes	15 (28.8%)	11 (42.3%)	4 (15.4%)	0.03
Cardiovascular disease	2 (3.8%)	1 (3.8%)	1 (3.8%)	1.0
Cerebrovascular diseases	3 (5.8%)	1 (3.8%)	2 (7.7%)	0.55
**Laboratory findings**				
Absolute neutrophil count (× 1000 cells per μL)	2.5 (0.9, 5.7)	2.8 (1.0, 6.7)	2.2 (0.9, 4.5)	0.60
Lymphocyte count (× 1000 cells per μL)	1.1 ± 0.7	1.2 ± 0.8	0.9 ± 0.6	0.31
Platelet count (× 1000 cells per μL)	44.0 (35.5, 69.0)	45.0 (40.3, 65.3)	41.5 (31.0, 73.3)	0.93
Hemoglobin concentration (g/L)	138.7 ± 24.7	134.2 ± 27.5	143.2 ± 21	0.19
Ferritin concentration (g/L)	9406.5 (3821.8, 18,885.8)	9442 (3584.3, 17,969.3)	9197 (4017.25, 19,718.5)	0.96
C-reactive protein (mg/L)	12.3 (1.9, 28.5)	14.8 (0.6, 38.0)	10.1 (3.6, 26.7)	0.88
Alanine aminotransferase (U/L)	124.5 (59.3, 176.8)	127.0 (57.5, 156.8)	122 (66.0, 207.5)	0.35
Aspartate aminotransferase (U/L)	319.0 (123.5, 587.3)	206.5 (104.3, 575.5)	422.0 (137.3, 660.8)	0.12
Blood urea nitrogen (mmol/L)	6.3 (4.0, 9.7)	5.0 (4.0, 7.7)	7.4 (3.9, 11.2)	0.10
Creatinine (µmol/L)	60.0 (51.3, 82.8)	59.5 (50.8, 76.0)	66.0 (51.8, 85.3)	0.37
Lactate dehydrogenase concentration (U/L)	1014.5 (558.0, 1930.3)	1168.5 (544.5, 1926.8)	883.0 (567.5, 2025.8)	0.86
Activated partial thromboplastin time (s)	60.2 ± 23.5	57.2 ± 21.7	63.1 ± 25.2	0.37
Galactomannan (pg/ml)	1.9 (0.4, 5.2)	1.9 (0.6, 4.9)	1.9 (0.3, 7.1)	0.99
SFTSV loads (log 10 copies/ml)	6.2 ± 1.7	6.2 ± 1.5	6.2 ± 1.8	0.90
Pneumonia	47 (90.4%)	24 (92.3%)	23 (88.5%)	0.64
Combined IPA	37 (71.2%)	19 (73.1%)	18 (69.2%)	0.76

Data are* n* (%), mean (SD), or median (IQR) unless otherwise stated. Data were assessed at baseline (the first day of hospitalization)

*Abbreviations*: *IPA* invasive pulmonary aspergillosis, *RUX* ruxolitinib treatment group, *HC* history control group

We also collected data on the use of combined treatments, including corticosteroids, immunoglobulin, antibacterial drugs, antifungal drugs, and blood component transfusions, in these two groups (Additional file 1: Table S4). There was no significant difference in the use of corticosteroids, immunoglobulin, antifungal drugs, or blood component transfusions between the two groups, and approximately 60% of patients in both groups received corticosteroids (65.4% in the RUX group and 57.7% in the HC group). The use of antibacterial drugs in the historical control group was significantly greater than that in the RUX group (*P* < 0.05).

### Primary and secondary outcomes

**Fig. 1:**
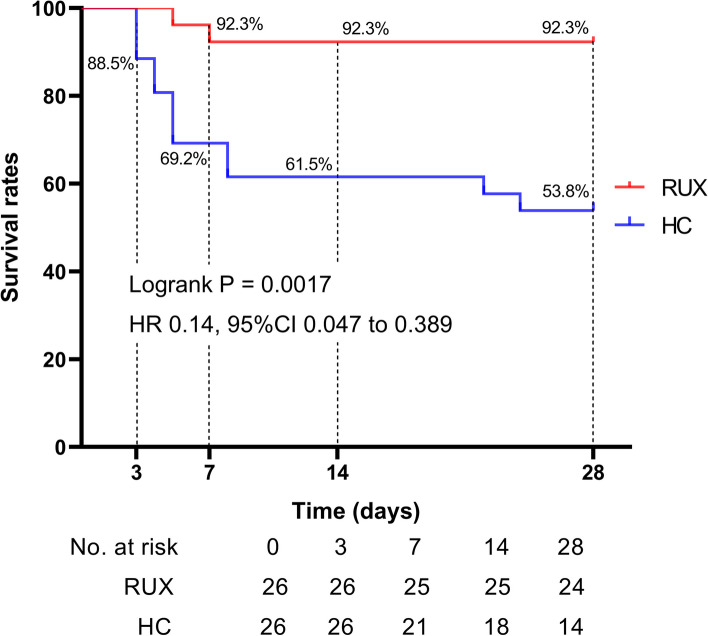
Kaplan–Meier curves for the ruxolitinib treatment effect on the probability of survival. All-cause mortality includes deaths potentially related with SFTS and deaths attributed to adverse events. *p* values were calculated from an unstratified log-rank test. Abbreviations: RUX, ruxolitinib group; HC, history control group; HR, hazard ratio

For the primary endpoint, 2 (7.7%) participants in the RUX group died of multiple organ failure within 7 days. The 28-day overall mortality rate was 7.7% in the RUX group and 46.2% in the HC group (*P* = 0.0017, log-rank test; Fig. 1), for a hazard ratio of 0.14 (95% CI 0.047–0.389).

Compared with those in the HC group, there were significantly fewer ICU admissions (15.4% vs 65.4%, *p* < 0.001) in the RUX group. The median hospitalization cost in the RUX group was 28,183.5 (IQR 15,315.3–54,763.8) Chinese yuan (CNY), which was significantly lower than that in the HC group (67,754.0 (IQR 40,990.8–99,404.5) CNY, *p* = 0.01). There were no significant differences in the duration of hospitalization between these two groups (Table 2).

**Table 2:** The proportion of ICU admissions, hospitalization costs, and stay in two groups

	**Total (*n* = 52)**	**RUX (*n* = 26)**	**HC (*n* = 26)**	***P*-value**
Hospitalization (days)	11.0 (7.0, 13.8)	10.5 (7.8, 13.3)	11.0 (6.0, 14.0)	0.73
ICU admissions (*n*, %)	21 (41.2%)	4 (15.4%)	17 (65.4%)	0.00
Hospital costs (CNY)	46,114.5 (19,262.25, 83,145.5)	28,183.5 (15,315.3, 54,763.8)	67,754.0 (40,990.8, 99,404.5)	0.01

Data are *n* (%), or median (IQR) unless otherwise stated

*Abbreviations*: *ICU* intensive care unit, *CNY* Chinese yuan, *RUX* ruxolitinib treatment group, *HC* history control group

**Fig. 2:**
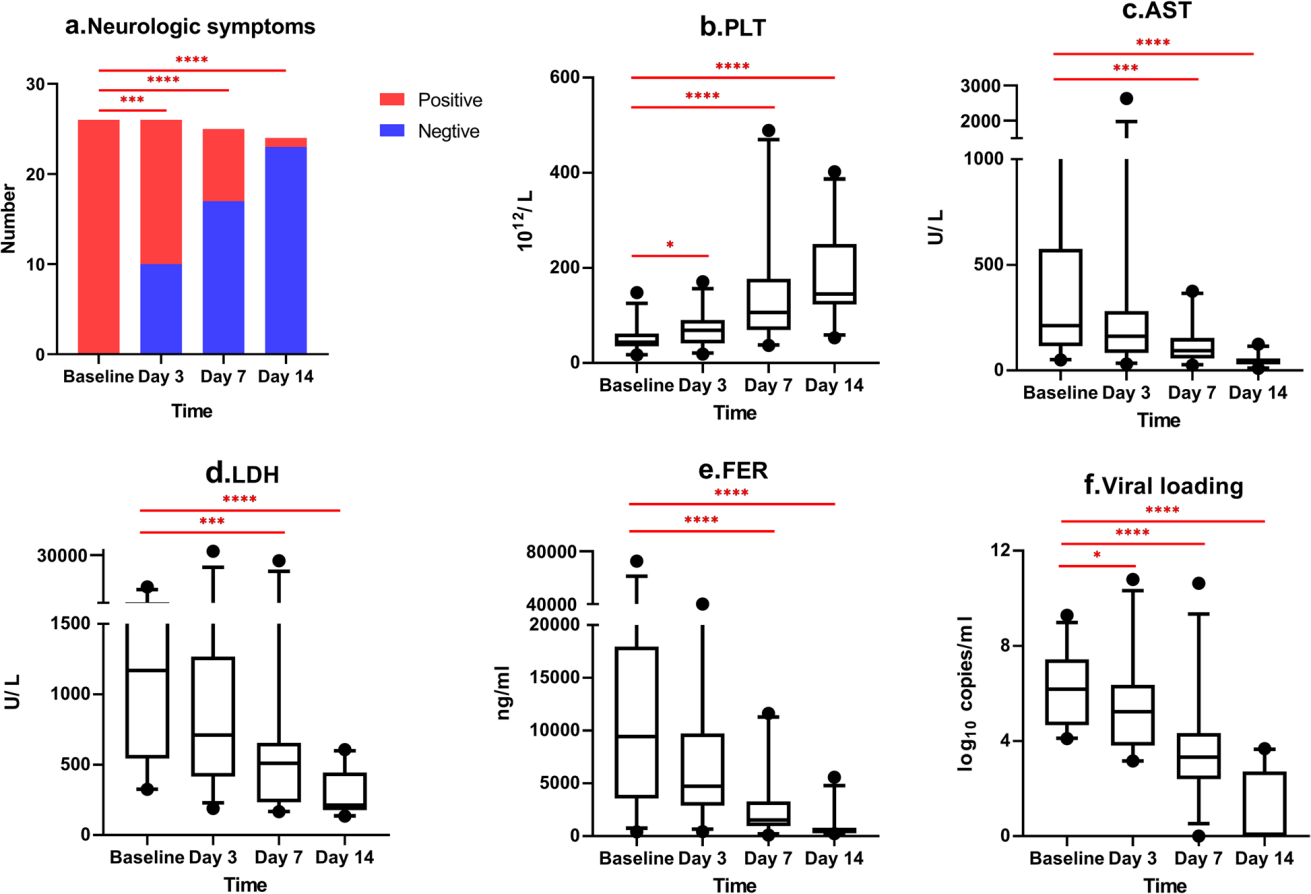
Improvements in neurologic symptoms and laboratory abnormalities of the RUX group. *: *p* < 0.05; ***: *p* < 0.001; ****: *p* < 0.0001. Abbreviations: PLT, platelet counts; AST, aspartate aminotransferase; LDH, lactate dehydrogenase; FER, ferritin

Substantial improvements in neurological symptoms and laboratory abnormalities were observed within 14 days in surviving participants in the RUX group (Fig. 2). We observed gradual remission of neurologic symptoms in the surviving participants over time, with 71% remission on day 7 and 96% remission on day 14 (Fig. 2a). Overall, the platelet counts increased gradually and approached normal at 1 week (Fig. 2b). Increased aspartate aminotransferase (AST), lactate dehydrogenase, and hyperferritinemia levels substantially improved within 2 weeks (Fig. 2c, d, and e). An absolute decrease in the serum SFTS viral load was observed (Fig. 2f).

### Safety

For safety endpoints, we evaluated clinical manifestations and selected laboratory parameters that were likely related to the adverse effects of ruxolitinib (Table 3). TRAEs developed in 6 patients (23.2%), including grade 3 in 2 patients (7.7%), and worsening in 8 patients (30.8%), including grade 3 in 5 patients (19.2%). In no case did they lead to drug discontinuation. Pneumonia was present at baseline in 24 patients, 3 of whom progressed within 1 week and one of whom improved at 2 weeks. One patient developed a secondary infection, a perianal subcutaneous abscess, which improved after 1 week of antibiotic treatment. There were 4 ICU admissions and 2 deaths in the RUX group, but no ICU admissions or deaths due to the toxicity of ruxolitinib.

**Table 3:** Treatment-related adverse events in the ruxolitinib treatment group

**Event or abnormalities**	**Before initiation of treatment, *n* (%)**	**Developed during treatment, *n* (%)**		**Worsened during treatment, *n* (%)**	
		**Any grade**	**Grade 3**	**Any grade**	**Grade 3**
**Any adverse events**		6 (23.2%)	2 (7.7%)	8 (30.8%)	5 (19.2%)
**Neutropenia**	12 (46.2%)	0	0	2 (7.7%)	1 (3.8%)
**Lymphopenia**	18 (69.2%)	2 (7.7%)	0	4 (15.4%)	2 (7.7%)
**Anemia**	5 (19.2%)	2 (7.7%)	0	1 (3.8%)	1 (3.8%)
**Thrombocytopenia**	25 (96.2%)	1 (3.8%)	0	3 (11.5%)	2 (7.7%)
**AST increased**	25 (96.2%)	0	0	5 (19.2%)	3 (11.5%)
**Bleeding**	6 (23.2%)	1 (3.8%)	1 (3.8%)	0	0
**Headache**	5 (19.2%)	2 (7.7%)	0	0	0
**Pneumonia**	24 (92.3%)	0	0	3 (11.5%)	0
**Other secondary infection**	3 (11.5%)	1 (3.8%)	1 (3.8%)	0	0

Data are *n* (%). Data were assessed from day 1 to day 28

*Abbreviations*: *AST* aspartate aminotransferase

### Exploratory laboratory analyses

We examined cytokine levels in the RUX group before ruxolitinib treatment. The median level of IL-6 is 58.8 (IQR 16.2–270.0) pg/ml, IL-8 is 41.4 (IQR 16.5–329.0) pg/ml, IL-10 is 47.0(IQR 9.9–113.7) pg/ml, and IFN-α is 17.7(IQR 2.5–105.5) pg/ml. IL-6, IL-10, and IFN-α were significantly elevated, with IL-6 being more pronounced. IFN-γ was elevated in only 19.2% of the participants. The delta cycle threshold (Ct) value of the IFI44L gene was 3.9 (IQR 3.1–4.8), which was significantly lower than that of the healthy control gene (8.53 delta Ct value), suggesting high expression of this gene in this group (Additional file 1: Fig. S2).

## Discussion

Our results suggest that ruxolitinib is tolerated and statistically associated with improvement of clinical outcome of severe SFTS. We found a lower risk of fatal outcomes with RUX treatment (7.7% vs. 46.2% in the HC group). Compared with those in the HC group, the ICU admission rate and hospitalization expenses of patients receiving ruxolitinib treatment significantly decreased. Rapid resolution of neurologic symptoms and laboratory abnormalities was observed. Although exacerbation of thrombocytopenia is one of the potential risks of ruxolitinib, we observed rapid improvement in thrombocytopenia in all survivors. Secondary infections are another potential risk factor for the use of ruxolitinib. A mild secondary infection was observed in one participant, which was similar to the treatment of COVID-19 with baricitinib [22]. A possible reason may be the short treatment course.

These preliminary findings are important, as SFTS is an emerging infectious disease associated with high mortality, particularly in patients with a clinical score greater than 8 points according to the clinical scoring model [9]. Cytokine storms and high viral loads are the two major prognostic indicators of SFTS. There is currently no specific therapy available for SFTS, and management primarily involves providing supportive care. Among antivirals, clinical studies of ribavirin for the treatment of SFTS have shown conflicting results [9, 23]. The efficacy and safety of favipiravir in the treatment of SFTS have been previously demonstrated [24]; however, additional evidence is needed for further verification. Corticosteroid treatment is often used to suppress cytokine storms in patients with SFTS. There are some case reports showing the effectiveness of corticosteroid treatment, and several retrospective cohort studies suggest the negative effects of corticosteroid therapy in severe SFTS [11, 25–28]. To the best of our knowledge, there are only two cases of immunomodulatory therapy for SFTS: one is tocilizumab therapy for nonfatal SFTS patients [29], and the other is ruxolitinib for severe SFTS [30]. Therefore, novel therapeutic strategies are needed.

To date, the underlying pathogenesis mechanisms of SFTS have remained unknown. A previous study reported that the cytokines IL-1RA, IL-6, IL-10, G-CSF, IP-10, and MCP-1 were elevated in SFTS patients and produced at robust levels in fatal cases [11]. Elevated cytokines such as IL-6, IL-10, and IFN-α were also observed in most participants in our study. Host cytokine storms during the acute phase of SFTS are the main cause of morbidity and mortality [10]. The IFI44L gene is a protein-coding gene associated with type I IFN responses [31, 32]. We observed that the IFI44L gene had a high expression level before ruxolitinib treatment (Additional file 1: Fig. S2).

These results indicate that the host type I interferon pathway was activated upon infection with SFTSV. Hence, JAK inhibitor treatment in the acute phase of SFTS to block the type I interferon pathway and cytokine storm was hypothesized to improve the outcome. In our study, ruxolitinib treatment started at a mean of 7.2 ± 1.5 days after symptom onset but started at more than 9 days in both deceased participants who suffered a fully activated cytokine storm and multiple-organ failure.

We hypothesized that the deaths of these two participants may be related to the late initiation of ruxolitinib treatment, and further research is needed to explore the ideal treatment timing.

The rarity and high mortality of SFTS have hampered the development of novel therapeutic strategies, including random controlled clinical trials. As a single-arm study, our study has limitations that prevent a full interpretation of the results. These limitations included the heterogeneity of combined treatments, the absence of a randomized control group, and the small sample size. Therefore, we matched the baseline characteristics of the HC group with those of the RUX group. Additionally, considering the possible impact of combined standard treatment, especially corticosteroid use, we compared corticosteroid use between the two groups and found no significant difference between them. In the RUX group, approximately 65% of patients received different doses (5 ~ 15 mg dexamethasone per day, or a corresponding dose of a systemic corticosteroids) of corticosteroids (mainly dexamethasone) for 3–5 days. The combination of ruxolitinib with corticosteroids, particularly dexamethasone, might have additive or synergistic effects on the treatment of patients with cytokine storms, as observed in hyperinflammation [33]and COVID-19 [22]. Further research is necessary to determine the optimal type, dosage, and course of corticosteroids. Although we balanced the confounding and misclassification of covariates such as gender, time from initial symptoms to hospitalization, clinical score within 6 days of onset, symptoms, and part of laboratory examinations between the RUX group and the HC group by PSM, we cannot completely rule out the effect of biases. As an exploratory trial, the sample size of our study is small, although this study shows the potential of ruxolitinib treatment to improve the outcomes, more research is needed in larger patient cohorts.

## Conclusions

Our findings indicate that ruxolitinib has the potential to increase the likelihood of survival as well as reduce the proportion of ICU hospitalization and being tolerated in severe SFTS. Furthermore, controlled clinical trials are necessary to establish the efficacy of ruxolitinib in treating SFTS.

### Supplementary Information

Additional file 1: Figure S1: Flow Diagram. Figure S2: Cytokines and IFI44L gene at baseline in ruxolitinib group. Table S1: The clinical scoring model based on age, and neurologic symptoms, and 4 laboratory indicators. Table S2: Propensity score matching methods for RUX and HC group. Table S3: Baseline characteristics before propensity score matching methods. Table S4: Combinations of standard of care in two groups.

## Data Availability

The data utilized in this study are available from the corresponding author on reasonable request.

## Abbreviations

AEs: Adverse events
AST: Aspartate aminotransferase
CNY: Chinese yuan
HC: Historical control
HR: Hazard ratio
ICU: Intensive care unit
IFI44L: Interferon-induced protein 44-like
IFN: Interferons
IFN-α: Interferon-α
IFN-γ: Interferon-γ
IL-10: Interleukin-10
IL-6: Interleukin-6
IL-8: Interleukin-8
IPA: Invasive pulmonary aspergillosis
IQR: Interquartile range
JAK: Janus kinase
RT-PCR: Reverse-transcriptase PCR
RUX: Ruxolitinib
SD: Standard deviations
SFTS: Severe fever with thrombocytopenia syndrome
SFTSV: SFTS virus
TRAEs: Treatment-related adverse events
