# Supplementary material for: Ruxolitinib plus standard of care in severe hospitalized adults with severe fever with thrombocytopenia syndrome (SFTS): an exploratory, single-arm trial
Source: BMC Med. 2024 May 20;22:204. doi: 10.1186/s12916-024-03421-z (PMC11103999; doi:10.1186/s12916-024-03421-z)
Supplement: Supplementary file 1 — Additional file 1: Figure S1: Flow Diagram. Figure S2: Cytokines and IFI44L gene at baseline in ruxolitinib group. Table S1: The clinical scoring model based on age, and neurologic symptoms, and 4 laboratory indicators. Table S2: Propensity score matching methods for RUX and HC group. Table S3: Baseline characteristics before propensity score matching methods. Table S4: Combinations of standard of care in two groups. [file 12916_2024_3421_MOESM1_ESM.docx]

**Ruxolitinib plus standard of care in severe hospitalized adults with severe fever with thrombocytopenia syndrome (SFTS): an exploratory, single-arm trial**

Sai Wen ^1^, Nannan Xu ^1^, Lianhui Zhao ^1^, Lulu Yang ^1^, Hui Yang ^1^, Caiyun Chang ^2^, Shanshan Wang ^1^, Chunmei Qu ^1^, Li Song ^1^, Wenlu Zou ^1^, Yishan He ^1^, Gang Wang ^1*^

1. Department of Infectious Disease, Qilu Hospital, Cheeloo College of Medicine, Shandong University, Jinan, 250012, Shandong, China.
2. Jinan Center for Disease Control and Prevention, Jinan, 250021, Shandong, China.

**Figure S1.** Flow Diagram

**Figure S2.** Cytokines and IFI44L gene at baseline in ruxolitinib group

**Table S1.** The clinical scoring model based on age, and neurologic symptoms, and 4 laboratory indicators

**Table S2.** Propensity score matching methods for RUX and HC group

**Table S3.** Baseline characteristics before propensity score matching methods

**Table S4.** Combinations of standard of care in two groups

**1.Eligibility Criteria**

**Inclusion criteria**

Patients with SFTS were included in accordance with the following inclusion criteria:

(1) Age ≥ 18 years.

(2) Laboratory-confirmed SFTS use the definition released by the Chinese Ministry of Health^[10]^: suspected diagnosed SFTS (an exposure history of previous field activities in SFTS pandemic areas or tick bites within 2 weeks before febrile symptom onset, acute fever with thrombocytopenia and/or leukopenia) with detection of SFTSV RNA by reverse-transcriptase PCR (RT-PCR).

(3) Hospitalized patients.

(4) Clinical score more than 8 points within 6 days of symptom onset^[9]^, see supplementary table 1 for details.

(5) Signed Informed consent.

**B. Exclusion criteria included:**

(1) Pregnancy or breastfeeding.

(2) Current treatment with interferon, tocilizumab or similar drugs during current SFTSV infection.

(3) History of hypersensitivity to any drugs or metabolites of similar chemical classes as ruxolitinib.

(4) Unlikely to survive longer than 24 hours from enrollment in the investigator’s judgment.

**C. Treatment suspension criteria included**:

(1) Voluntary decision of the patient.

(2) Treating physician's decision to discontinue the treatment.

(3) Development of ruxolitinib allergy.

**2. SFTSV detection and quantitation by real-time PCR**

A total of 200 μl of serum was used for RNA extraction via the RNeasy Mini Kit (Qiagen, Germany). The SFTSV nucleic acid tests were performed using an SFTSV Real Time RT-PCR Kit (Daan gene, Guangzhou, China) according to the manufacturer’s protocol. The reaction parameters were 50℃ for 15 min, 95℃ for 15 min, 45 cycles of 94℃ for 15 s and 55℃ for 45 s, and then 40℃ for 10 s. The cut-off cycle threshold (Ct) value was set at 35 cycles.

**3.Serum cytokines (IL-6, IL-8, IL-10, IFN-α, IFN-γ) detection and quantitation by** **immunofluorescence assay.**

The serum cytokines tests were performed using immunofluorescence assay (Sakkibio, Nanchang, China) and BD FACSCantoll flow cytometer system (BD, Shanghai China) according to the manufacturer’s protocol.

**4. IFI44L transcript detection and quantitation by real-time RT-PCR**

**A.RNA extraction and reverse transcription**

Blood samples (1-2 ml each in volume) were drawn into EDTA-anticoagulation tubes concurrently with samples for routine clinical testing. Within 2 hours after collection, the blood sample was treated with TRIzol LS Reagent (Invitrogen) for subsequent RNA extraction. Total RNA was extracted using a Direct-zolTM RNA MiniPrep kit (ZYMO RESEARCH CORP) from the processed whole blood sample. 300 ng RNA was added to a 20-μl mixture containing Random Primer p(dN)6, reaction buffer, RNase Inhibitor, dNTP Mix, M-MuLV RT, and RNase free ddH2O using a reverse-transcription kit (Sangon Biotech Corp) with the following cycling profile: 65℃ for 5 min, 2 min in an ice bath, 25℃ for 10 min, 50℃ for 30 min, and 85℃ for 5 min and a hold at 4℃. The obtained cDNA was stored at -80℃ for further detection.

**B. Real-time PCR**

A TaqMan PCR kit and the primers for target genes were purchased from Applied Biosystems, including IFI44L (Hs00915292_m1) and the internal control ACTB (Hs01060665_g1). Reactions were performed in a 20-ul reaction volume in TaqMan Fast qPCR Master Mix (Sangon Biotech Corp) using Hongshi SLAN96P platform. Single-gene single-channel dual-repetitive amplification was performed using a fluorescent quantitation PCR instrument. The PCR cycle was 50℃ for 2 min, 95℃ for 10 min and 36 cycles of 95℃ for 15 s and 60℃ for 1 min. Delta Ct method was used to calculate relative mRNA expression.

**Figure S1. Flow Diagram**


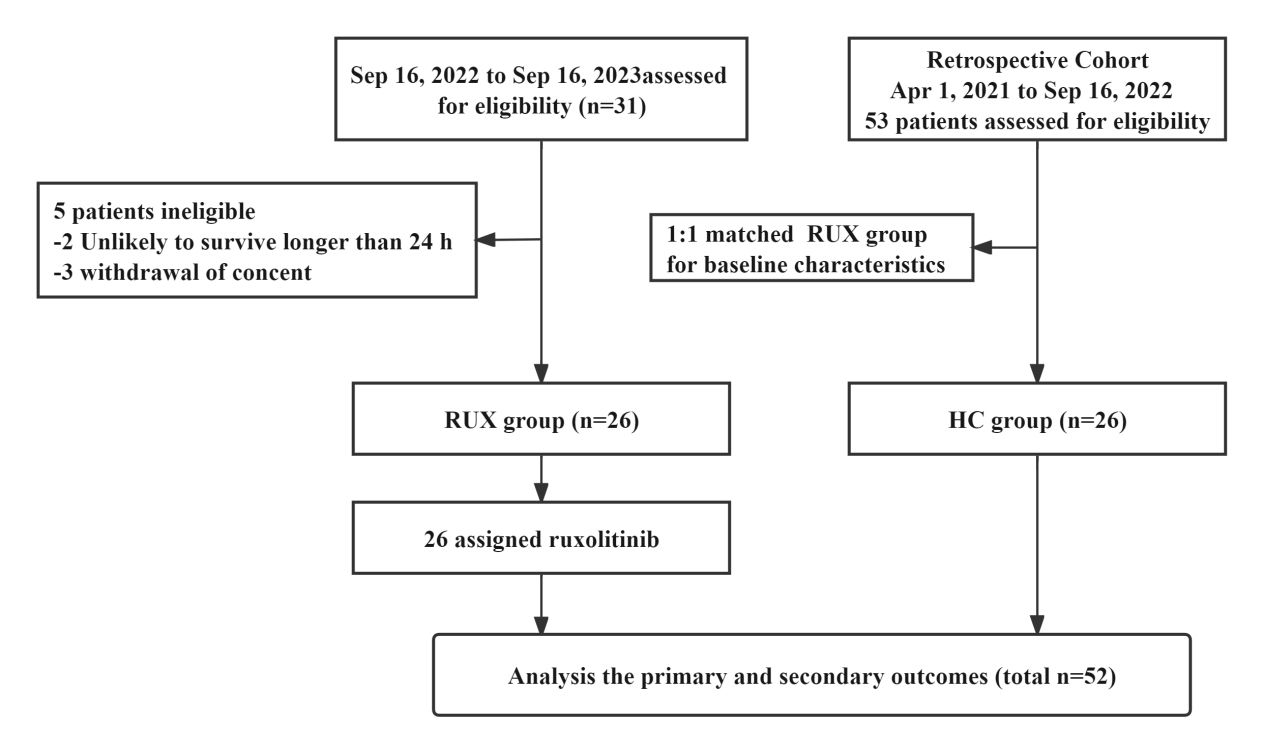


Abbreviations: RUX: ruxolitinib group; HC: history control group.

**Figure S2. Cytokines and IFI44L gene at baseline in ruxolitinib group.**


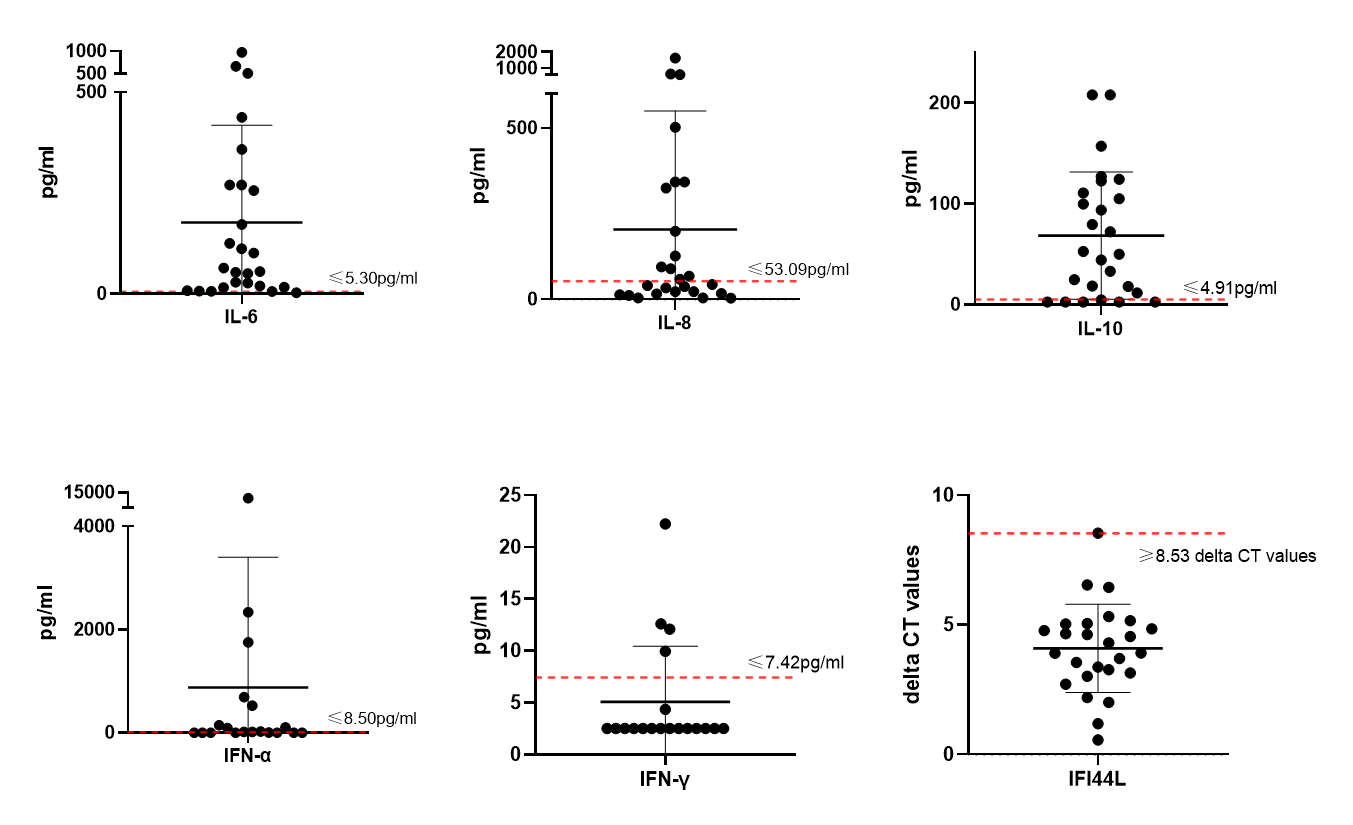


Abbreviations: IL-6: interleukin-6; IL-8: interleukin-8; IL-10: interleukin-10; IFN-α: interferon-α; IFN-γ: interferon-γ; IFI44L: transcripts of the interferon (IFN)-induced protein 44-like (IFI44L) gene.

**Table S1. The clinical scoring model based on age, and neurologic symptoms, and 4 laboratory indicators.**

| **Variable** | **range** | **Score** |
| --- | --- | --- |
| Age, years | <60 | 1 |
|  | 60-70 | 2 |
|  | ≥70 | 3 |
| Neurologic symptoms, no. | 0 | 1 |
|  | 1 | 2 |
|  | ≥2 | 3 |
| Lactate dehydrogenase, U/L | ＜600 | 1 |
|  | 600-1000 | 2 |
|  | ≥1000 | 3 |
| Aspartate aminotransferase, U/L | ＜200 | 1 |
|  | 200-4000 | 2 |
|  | ≥4000 | 3 |
| Blood urea nitrogen, mmol/L | ＜7.15 | 1 |
|  | 7.15-9 | 2 |
|  | ≥9 | 3 |
| Neutrophil percentage, % | ＜70 | 1 |
|  | 70-80 | 2 |
|  | ≥80 | 3 |

**Table S2. Propensity score matching methods for RUX and HC group.**

| Variables in logistics regression | B | P | Exp(B) |
| --- | --- | --- | --- |
| Gender | 0.430 | 0.473 | 1.537 |
| Time from initial symptoms to hospitalization | 0.552 | 0.007 | 1.737 |
| Clinical score within 6 days of onset | 0.155 | 0.431 | 1.167 |
| Gastrointestinal symptoms | 1.089 | 0.261 | 2.971 |
| Respiratory tract symptoms | -0.563 | 0.410 | 0.569 |
| Hemorrhage symptoms | -0.979 | 0.290 | 0.376 |
| Pre-existing comorbidity | -0.175 | 0.779 | 0.839 |
| platelet count | -0.008 | 0.451 | 0.992 |
| ferritin concentration | 0.000 | 0.852 | 1.000 |
| c-reactive protein | 0.001 | 0.892 | 1.001 |
| Creatinine(umol/L) | 0.008 | 0.294 | 1.008 |
| alanine aminotransferase | 0.001 | 0.650 | 1.001 |
| Match types | exact match | 0.000 |  |
|  | fuzzy matching | 26.000 |  |
| PSSS | 0.020 | | |
| matching attempt count | 686.000 | | |
| incremental rejection percentage | 97.376 | | |

**Table S3. Baseline characteristics before propensity score matching methods.**

|  | **Total**  **(N=79)** | **RUX**  **（n=26）** | **HC**  **（n=53）** | **P-value** |
| --- | --- | --- | --- | --- |
| **General** |  |  |  |  |
| Gender, male (n, %) | 44 (55.7%) | 15 (57.7%) | 29 (54.7%) | 0.77 |
| Age(years) | 64.1 ± 6.8 | 65.8 ± 8.7 | 63.6 ± 7.1 | 0.21 |
| Time from initial symptoms to hospitalization(days) | 8.0 ± 1.5 | 7.2 ± 1.5 | 8.4 ± 1.5 | 0.00 |
| **Clinical parameters** |  |  |  |  |
| Body temperature (℃) | 38.8 ± 0.6 | 38.8 ± 0.5 | 38.8 ± 0.6 | 0.37 |
| Gastrointestinal symptoms | 26 (32.9%) | 8 (30.8%) | 18 (34.0%) | 0.64 |
| Respiratory tract symptoms | 31 (39.2%) | 11 (42.3%) | 20 (37.7%) | 0.73 |
| Neurologic symptoms | 77 (97.5%) | 26 (100%) | 51 (96.2%) | 0.81 |
| Hemorrhage symptoms | 21 (26.6%) | 6 (23.1%) | 15 (28.3%) | 0.61 |
| Clinical score within 6 days of onset | 12.9 ± 1.7 | 12.2 ± 1.7 | 12.9 ± 1.8 | 0.41 |
| **Pre-existing comorbidity** | 51 (64.6%) | 17 (65.4%) | 34 (64.2%) | 0.79 |
| Hypertension | 22 (27.8%) | 6 (23.1%) | 16 (30.2%) | 0.57 |
| Diabetes | 20 (25.3%) | 11 (42.3%) | 9 (17.0%) | 0.03 |
| Cardiovascular disease | 5 (6.3%) | 1 (3.8%) | 4 (7.5%) | 0.42 |
| Cerebrovascular diseases | 5 (6.3%) | 1(3.8%) | 4 (7.5%) | 0.42 |
| **Laboratory findings** |  |  |  |  |
| Absolute neutrophil count (×1000 cells per μL) | 1.6 (0.6, 4.3) | 2.8 (1.0, 6.7) | 1.2 (0.5, 2.4) | 0.01 |
| Lymphocytes count (×1000 cells per μL) | 0.8 (0.4, 1.5) | 1.2 ± 0.8 | 0.9 ± 0.6 | 0.14 |
| Platelet count (×1000 cells per μL) | 38.0 (27.0, 48.0) | 45.0 (40.3, 65.3) | 33.3 (27.0, 41.5) | 0.00 |
| Hemoglobin concentration(g/L) | 138.9 ± 20.4 | 134.2 ± 27.5 | 140.0 ± 18.9 | 0.67 |
| Ferritin concentration(g/L) | 9756.0 (5621.8, 16273.8) | 9442 (3584.3, 17969.3) | 9740 (5092.5, 17809.5) | 0.79 |
| C-reactive protein(mg/L) | 23.7 (8.0, 29.2) | 14.8 (0.6, 38.0) | 24.3 (9.2, 28.4) | 0.47 |
| Alanine aminotransferase (U/L) | 130.0 (68.0, 176.0) | 127.0 (57.5, 156.8) | 130.0 (67.0, 194.0) | 0.61 |
| Aspartate aminotransferase (U/L) | 235.8 (122.0, 560.0) | 206.5 (104.3, 575.5) | 299.0 (120.0, 547.5) | 0.51 |
| Blood urea nitrogen(mmol/L) | 5.3 (4.1, 7.9) | 5.0 (4.0, 7.7) | 5.3 (4.0, 7.9) | 0.96 |
| Creatinine(umol/L) | 63.0 (53.0, 81.0) | 59.5 (50.8, 76.0) | 66.0 (51.5, 83.5) | 0.44 |
| Lactate dehydrogenase concentration(U/L) | 965.0 (546.0, 1646.0) | 1168.5 (544.5, 1926.8) | 867.0 (564.0, 1368.5) | 0.32 |
| Activated partial thromboplastin time(s) | 54.3 ± 21.7 | 57.2 ± 21.7 | 52.0 ± 21.9 | 0.09 |
| Galactomannan(pg/ml) | 1.6 (0.4, 4.6) | 1.9 (0.6, 4.9) | 1.3 (0.3, 3.6) | 0.17 |
| SFTSV loads (log 10 copies/ml) | 6.2 ± 1.7 | 6.2±1.5 | 6.1±1.8 | 0.76 |
| Pneumonia | 74 (88.1%) | 24 (92.3%) | 45 (84.9%) | 0.24 |
| Combined IPA | 58 (69.0%) | 19 (73.1%) | 36 (67.9%) | 0.77 |

Abbreviations: RUX: ruxolitinib group; HC: history control group. IPA: invasive pulmonary aspergillosis.

**Table S4. Combinations of standard of care in two groups.**

|  | **Total (n=52)** | **RUX（n=26）** | **HC（n=26）** | **P-value** |
| --- | --- | --- | --- | --- |
| Corticosteroid | 32 (61.5%) | 17 (65.4%) | 15 (57.7%) | 0.57 |
| Immunoglobulin | 5(9.6%) | 1 (3.8%) | 4 (15.4%) | 0.09 |
| Antibacterial drugs | 38 (73.1%) | 14 (53.8%) | 24 (92.3%) | 0.002 |
| Antifungal drugs | 46 (88.5%) | 24 (92.3%) | 22 (84.6%) | 0.39 |
| Blood components transfusion | 17 (32.7%) | 7 (26.9%) | 10 (38.5%) | 0.38 |

Data are n (%); n is number of participants. Data were assessed from days 1 to 28.

Abbreviations: RUX: ruxolitinib group; HC: history control group.
